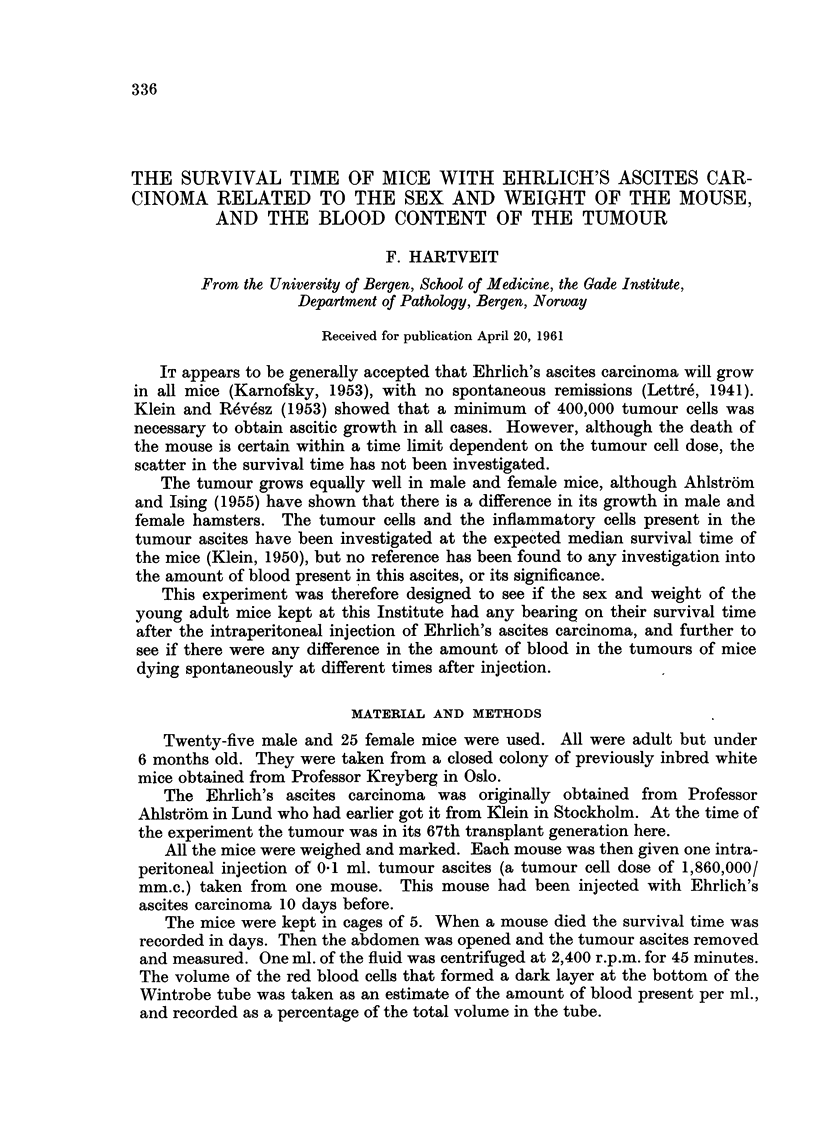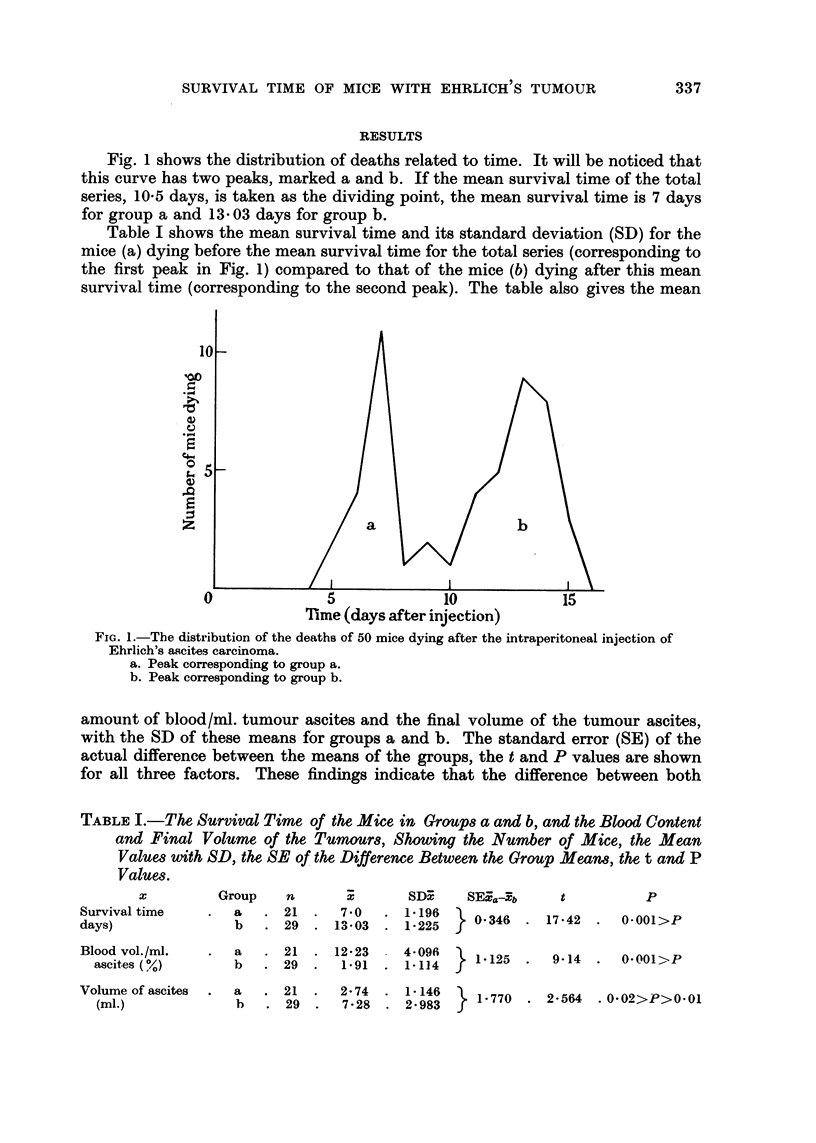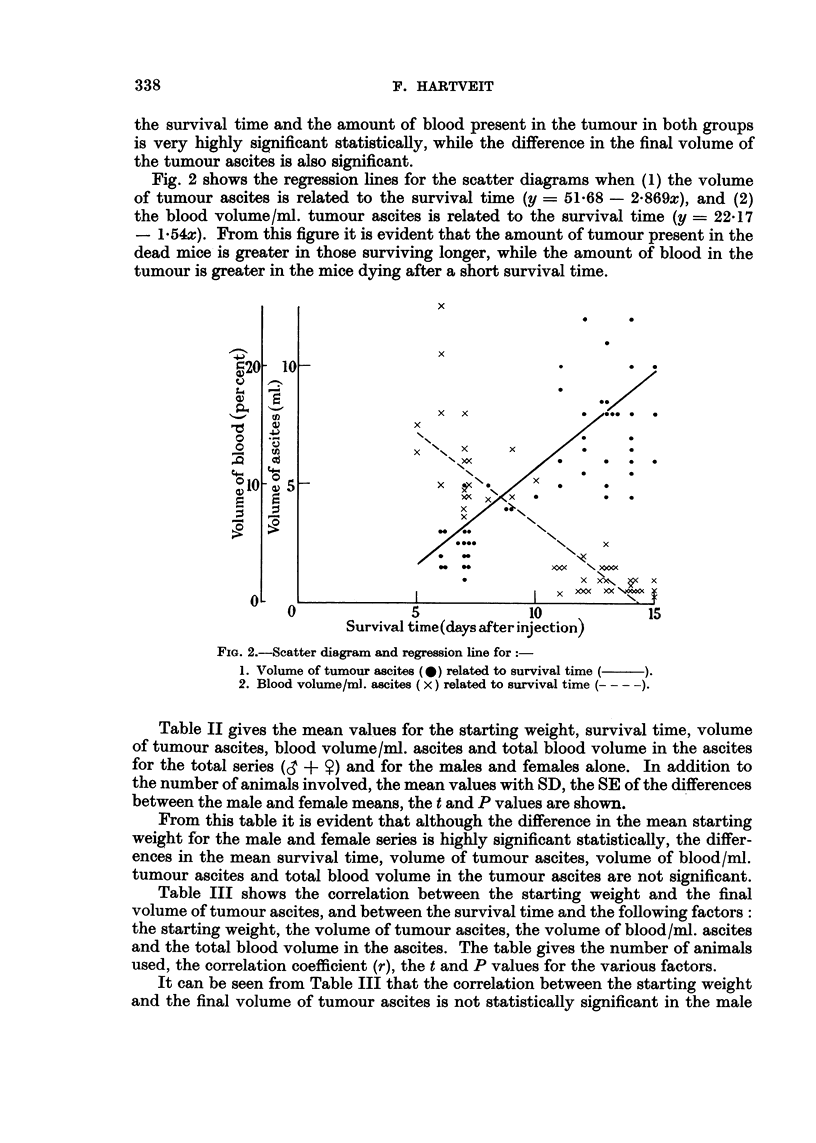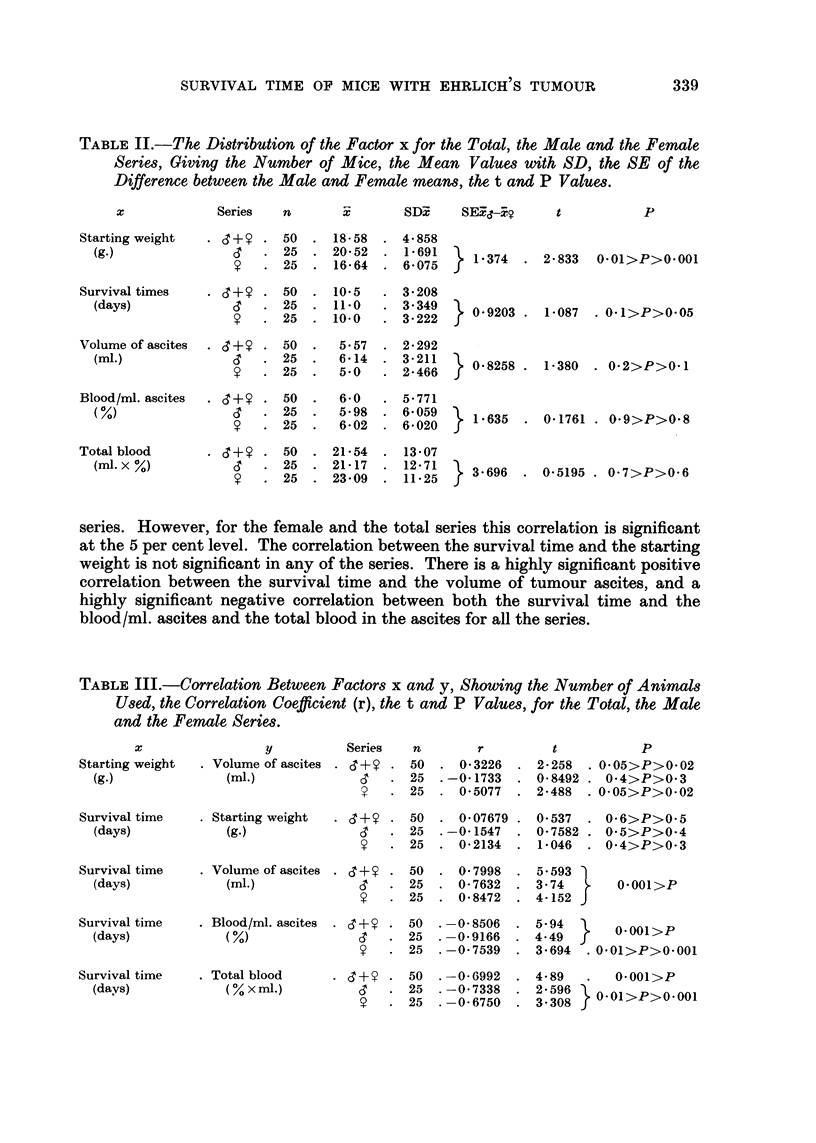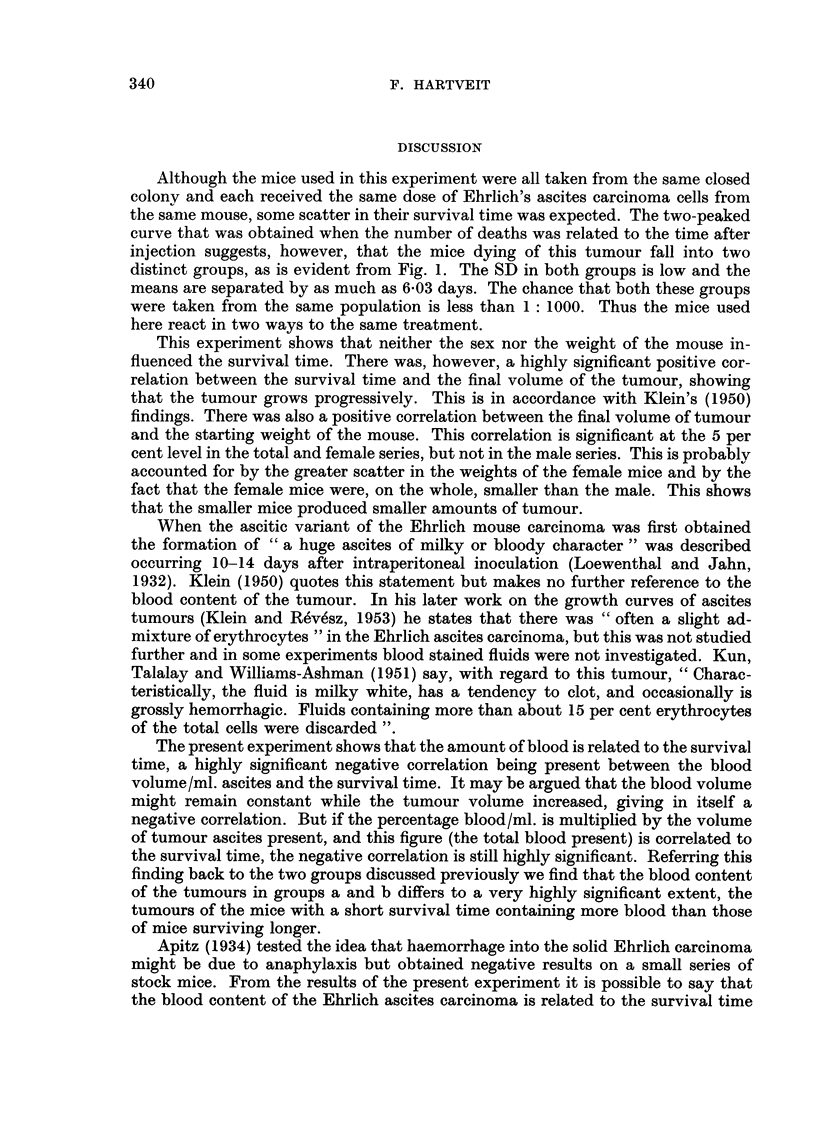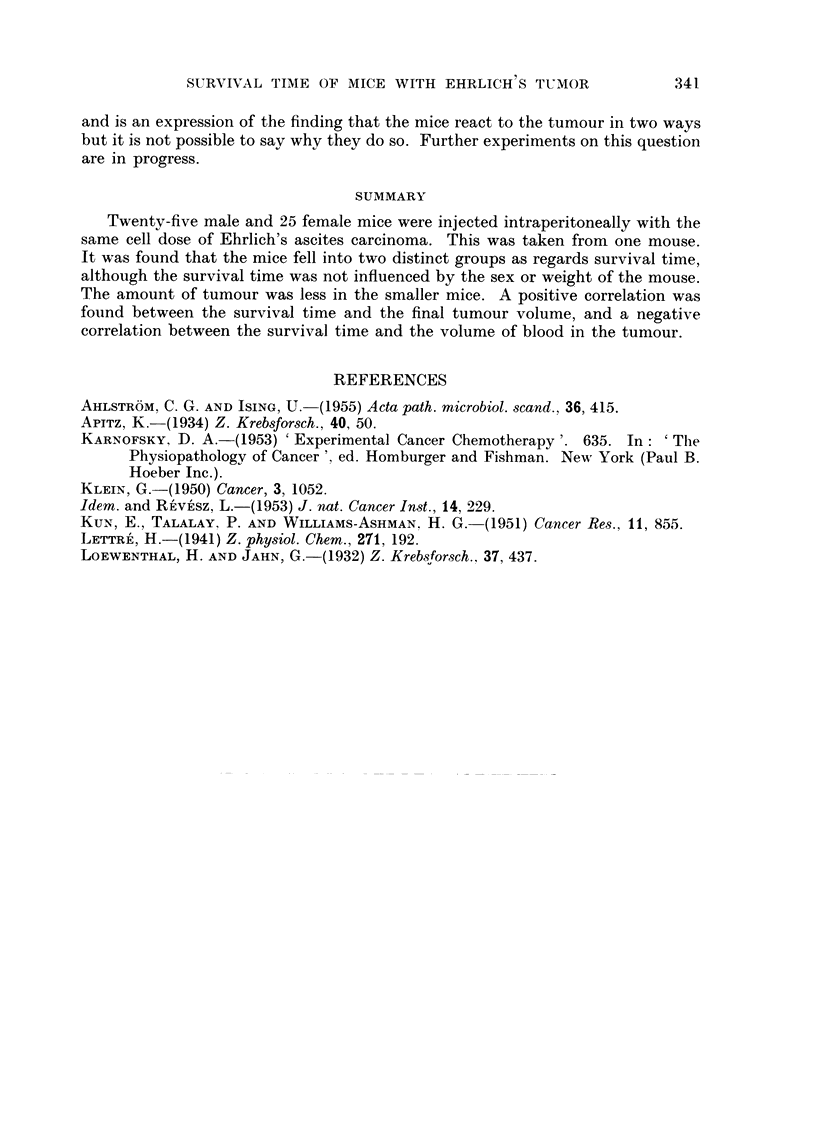# The Survival Time of Mice with Ehrlich's Ascites Carcinoma related to the Sex and Weight of the Mouse, and the Blood Content of the Tumour

**DOI:** 10.1038/bjc.1961.42

**Published:** 1961-06

**Authors:** F. Hartveit


### THE SURVIVAL TIME OF MICE WITH EHRLICH'S ASCITES CARCINOMA RELATED TO THE SEX AND WEIGHT OF THE MOUSE, AND THE BLOOD CONTENT OF THE TUMOUR

F. HARTVEIT

*From the University of Bergen, School of Medicine, the Gade Institute, Department of Pathology, Bergen, Norway*

Received for publication April 20, 1961

IT appears to be generally accepted that Ehrlich's ascites carcinoma will grow in all mice (Karnofsky, 1953), with no spontaneous remissions (Lettré, 1941). Klein and Révész (1953) showed that a minimum of 400,000 tumour cells was necessary to obtain ascitic growth in all cases. However, although the death of the mouse is certain within a time limit dependent on the tumour cell dose, the scatter in the survival time has not been investigated.

The tumour grows equally well in male and female mice, although Ahlström and Ising (1955) have shown that there is a difference in its growth in male and female hamsters. The tumour cells and the inflammatory cells present in the tumour ascites have been investigated at the expected median survival time of the mice (Klein, 1950), but no reference has been found to any investigation into the amount of blood present in this ascites, or its significance.

This experiment was therefore designed to see if the sex and weight of the young adult mice kept at this Institute had any bearing on their survival time after the intraperitoneal injection of Ehrlich's ascites carcinoma, and further to see if there were any difference in the amount of blood in the tumours of mice dying spontaneously at different times after injection.

### MATERIAL AND METHODS

Twenty-five male and 25 female mice were used. All were adult but under 6 months old. They were taken from a closed colony of previously inbred white mice obtained from Professor Kreyberg in Oslo.

The Ehrlich's ascites carcinoma was originally obtained from Professor Ahlström in Lund who had earlier got it from Klein in Stockholm. At the time of the experiment the tumour was in its 67th transplant generation here.

All the mice were weighed and marked. Each mouse was then given one intraperitoneal injection of 0·1 ml. tumour ascites (a tumour cell dose of 1,860,000/mm.c.) taken from one mouse. This mouse had been injected with Ehrlich's ascites carcinoma 10 days before.

The mice were kept in cages of 5. When a mouse died the survival time was recorded in days. Then the abdomen was opened and the tumour ascites removed and measured. One ml. of the fluid was centrifuged at 2,400 r.p.m. for 45 minutes. The volume of the red blood cells that formed a dark layer at the bottom of the Wintrobe tube was taken as an estimate of the amount of blood present per ml., and recorded as a percentage of the total volume in the tube.

### RESULTS

Fig. 1 shows the distribution of deaths related to time. It will be noticed that this curve has two peaks, marked a and b. If the mean survival time of the total series, 10·5 days, is taken as the dividing point, the mean survival time is 7 days for group a and 13·03 days for group b.

Table I shows the mean survival time and its standard deviation (SD) for the mice (a) dying before the mean survival time for the total series (corresponding to the first peak in Fig. 1) compared to that of the mice (*b*) dying after this mean survival time (corresponding to the second peak). The table also gives the mean

FIG. 1.—The distribution of the deaths of 50 mice dying after the intraperitoneal injection of Ehrlich's ascites carcinoma.
a. Peak corresponding to group a.
b. Peak corresponding to group b.

amount of blood/ml. tumour ascites and the final volume of the tumour ascites, with the SD of these means for groups a and b. The standard error (SE) of the actual difference between the means of the groups, the *t* and *P* values are shown for all three factors. These findings indicate that the difference between both

TABLE I.—*The Survival Time of the Mice in Groups a and b, and the Blood Content and Final Volume of the Tumours, Showing the Number of Mice, the Mean Values with SD, the SE of the Difference Between the Group Means, the* t *and* P *Values.*

| $x$ | Group | $n$ | $\bar{x}$ | SD$\bar{x}$ | SE$\bar{x}_a - \bar{x}_b$ | $t$ | $P$ |
|---|---|---|---|---|---|---|---|
| Survival time days) | a | 21 | 7·0 | 1·196 | 0·346 | 17·42 | $0\cdot001>P$ |
| | b | 29 | 13·03 | 1·225 | | | |
| Blood vol./ml. ascites (%) | a | 21 | 12·23 | 4·096 | 1·125 | 9·14 | $0\cdot001>P$ |
| | b | 29 | 1·91 | 1·114 | | | |
| Volume of ascites (ml.) | a | 21 | 2·74 | 1·146 | 1·770 | 2·564 | $0\cdot02>P>0\cdot01$ |
| | b | 29 | 7·28 | 2·983 | | | |

the survival time and the amount of blood present in the tumour in both groups is very highly significant statistically, while the difference in the final volume of the tumour ascites is also significant.

Fig. 2 shows the regression lines for the scatter diagrams when (1) the volume of tumour ascites is related to the survival time ($y = 51{\cdot}68 - 2{\cdot}869x$), and (2) the blood volume/ml. tumour ascites is related to the survival time ($y = 22{\cdot}17 - 1{\cdot}54x$). From this figure it is evident that the amount of tumour present in the dead mice is greater in those surviving longer, while the amount of blood in the tumour is greater in the mice dying after a short survival time.

Fig. 2.—Scatter diagram and regression line for :—

1. Volume of tumour ascites (●) related to survival time (———).
2. Blood volume/ml. ascites (×) related to survival time (- - - -).

Table II gives the mean values for the starting weight, survival time, volume of tumour ascites, blood volume/ml. ascites and total blood volume in the ascites for the total series (♂ + ♀) and for the males and females alone. In addition to the number of animals involved, the mean values with SD, the SE of the differences between the male and female means, the $t$ and $P$ values are shown.

From this table it is evident that although the difference in the mean starting weight for the male and female series is highly significant statistically, the differences in the mean survival time, volume of tumour ascites, volume of blood/ml. tumour ascites and total blood volume in the tumour ascites are not significant.

Table III shows the correlation between the starting weight and the final volume of tumour ascites, and between the survival time and the following factors : the starting weight, the volume of tumour ascites, the volume of blood/ml. ascites and the total blood volume in the ascites. The table gives the number of animals used, the correlation coefficient ($r$), the $t$ and $P$ values for the various factors.

It can be seen from Table III that the correlation between the starting weight and the final volume of tumour ascites is not statistically significant in the male

TABLE II.—*The Distribution of the Factor* x *for the Total, the Male and the Female Series, Giving the Number of Mice, the Mean Values with SD, the SE of the Difference between the Male and Female means, the* t *and* P *Values.*

| $x$ | Series | $n$ | $\bar{x}$ | SD$\bar{x}$ | SE$\bar{x}_{♂}-\bar{x}_{♀}$ | $t$ | $P$ |
|---|---|---|---|---|---|---|---|
| Starting weight (g.) | ♂+♀ | 50 | 18·58 | 4·858 | | | |
| | ♂ | 25 | 20·52 | 1·691 | 1·374 | 2·833 | $0·01>P>0·001$ |
| | ♀ | 25 | 16·64 | 6·075 | | | |
| Survival times (days) | ♂+♀ | 50 | 10·5 | 3·208 | | | |
| | ♂ | 25 | 11·0 | 3·349 | 0·9203 | 1·087 | $0·1>P>0·05$ |
| | ♀ | 25 | 10·0 | 3·222 | | | |
| Volume of ascites (ml.) | ♂+♀ | 50 | 5·57 | 2·292 | | | |
| | ♂ | 25 | 6·14 | 3·211 | 0·8258 | 1·380 | $0·2>P>0·1$ |
| | ♀ | 25 | 5·0 | 2·466 | | | |
| Blood/ml. ascites (%) | ♂+♀ | 50 | 6·0 | 5·771 | | | |
| | ♂ | 25 | 5·98 | 6·059 | 1·635 | 0·1761 | $0·9>P>0·8$ |
| | ♀ | 25 | 6·02 | 6·020 | | | |
| Total blood (ml. × %) | ♂+♀ | 50 | 21·54 | 13·07 | | | |
| | ♂ | 25 | 21·17 | 12·71 | 3·696 | 0·5195 | $0·7>P>0·6$ |
| | ♀ | 25 | 23·09 | 11·25 | | | |

series. However, for the female and the total series this correlation is significant at the 5 per cent level. The correlation between the survival time and the starting weight is not significant in any of the series. There is a highly significant positive correlation between the survival time and the volume of tumour ascites, and a highly significant negative correlation between both the survival time and the blood/ml. ascites and the total blood in the ascites for all the series.

TABLE III.—*Correlation Between Factors* x *and* y, *Showing the Number of Animals Used, the Correlation Coefficient* (r), *the* t *and* P *Values, for the Total, the Male and the Female Series.*

| $x$ | $y$ | Series | $n$ | $r$ | $t$ | $P$ |
|---|---|---|---|---|---|---|
| Starting weight (g.) | Volume of ascites (ml.) | ♂+♀ | 50 | 0·3226 | 2·258 | $0·05>P>0·02$ |
| | | ♂ | 25 | −0·1733 | 0·8492 | $0·4>P>0·3$ |
| | | ♀ | 25 | 0·5077 | 2·488 | $0·05>P>0·02$ |
| Survival time (days) | Starting weight (g.) | ♂+♀ | 50 | 0·07679 | 0·537 | $0·6>P>0·5$ |
| | | ♂ | 25 | −0·1547 | 0·7582 | $0·5>P>0·4$ |
| | | ♀ | 25 | 0·2134 | 1·046 | $0·4>P>0·3$ |
| Survival time (days) | Volume of ascites (ml.) | ♂+♀ | 50 | 0·7998 | 5·593 | |
| | | ♂ | 25 | 0·7632 | 3·74 | $0·001>P$ |
| | | ♀ | 25 | 0·8472 | 4·152 | |
| Survival time (days) | Blood/ml. ascites (%) | ♂+♀ | 50 | −0·8506 | 5·94 | $0·001>P$ |
| | | ♂ | 25 | −0·9166 | 4·49 | |
| | | ♀ | 25 | −0·7539 | 3·694 | $0·01>P>0·001$ |
| Survival time (days) | Total blood (% × ml.) | ♂+♀ | 50 | −0·6992 | 4·89 | $0·001>P$ |
| | | ♂ | 25 | −0·7338 | 2·596 | $0·01>P>0·001$ |
| | | ♀ | 25 | −0·6750 | 3·308 | |

### DISCUSSION

Although the mice used in this experiment were all taken from the same closed colony and each received the same dose of Ehrlich's ascites carcinoma cells from the same mouse, some scatter in their survival time was expected. The two-peaked curve that was obtained when the number of deaths was related to the time after injection suggests, however, that the mice dying of this tumour fall into two distinct groups, as is evident from Fig. 1. The SD in both groups is low and the means are separated by as much as 6·03 days. The chance that both these groups were taken from the same population is less than 1 : 1000. Thus the mice used here react in two ways to the same treatment.

This experiment shows that neither the sex nor the weight of the mouse influenced the survival time. There was, however, a highly significant positive correlation between the survival time and the final volume of the tumour, showing that the tumour grows progressively. This is in accordance with Klein's (1950) findings. There was also a positive correlation between the final volume of tumour and the starting weight of the mouse. This correlation is significant at the 5 per cent level in the total and female series, but not in the male series. This is probably accounted for by the greater scatter in the weights of the female mice and by the fact that the female mice were, on the whole, smaller than the male. This shows that the smaller mice produced smaller amounts of tumour.

When the ascitic variant of the Ehrlich mouse carcinoma was first obtained the formation of " a huge ascites of milky or bloody character " was described occurring 10–14 days after intraperitoneal inoculation (Loewenthal and Jahn, 1932). Klein (1950) quotes this statement but makes no further reference to the blood content of the tumour. In his later work on the growth curves of ascites tumours (Klein and Révész, 1953) he states that there was " often a slight admixture of erythrocytes " in the Ehrlich ascites carcinoma, but this was not studied further and in some experiments blood stained fluids were not investigated. Kun, Talalay and Williams-Ashman (1951) say, with regard to this tumour, " Characteristically, the fluid is milky white, has a tendency to clot, and occasionally is grossly hemorrhagic. Fluids containing more than about 15 per cent erythrocytes of the total cells were discarded ".

The present experiment shows that the amount of blood is related to the survival time, a highly significant negative correlation being present between the blood volume/ml. ascites and the survival time. It may be argued that the blood volume might remain constant while the tumour volume increased, giving in itself a negative correlation. But if the percentage blood/ml. is multiplied by the volume of tumour ascites present, and this figure (the total blood present) is correlated to the survival time, the negative correlation is still highly significant. Referring this finding back to the two groups discussed previously we find that the blood content of the tumours in groups a and b differs to a very highly significant extent, the tumours of the mice with a short survival time containing more blood than those of mice surviving longer.

Apitz (1934) tested the idea that haemorrhage into the solid Ehrlich carcinoma might be due to anaphylaxis but obtained negative results on a small series of stock mice. From the results of the present experiment it is possible to say that the blood content of the Ehrlich ascites carcinoma is related to the survival time and is an expression of the finding that the mice react to the tumour in two ways but it is not possible to say why they do so. Further experiments on this question are in progress.

### SUMMARY

Twenty-five male and 25 female mice were injected intraperitoneally with the same cell dose of Ehrlich's ascites carcinoma. This was taken from one mouse. It was found that the mice fell into two distinct groups as regards survival time, although the survival time was not influenced by the sex or weight of the mouse. The amount of tumour was less in the smaller mice. A positive correlation was found between the survival time and the final tumour volume, and a negative correlation between the survival time and the volume of blood in the tumour.

### REFERENCES

AHLSTRÖM, C. G. AND ISING, U.—(1955) *Acta path. microbiol. scand.*, **36**, 415.
APITZ, K.—(1934) *Z. Krebsforsch.*, **40**, 50.
KARNOFSKY, D. A.—(1953) ' Experimental Cancer Chemotherapy '. 635. In : ' The Physiopathology of Cancer ', ed. Homburger and Fishman. New York (Paul B. Hoeber Inc.).
KLEIN, G.—(1950) *Cancer*, **3**, 1052.
*Idem.* and RÉVÉSZ, L.—(1953) *J. nat. Cancer Inst.*, **14**, 229.
KUN, E., TALALAY, P. AND WILLIAMS-ASHMAN, H. G.—(1951) *Cancer Res.*, **11**, 855.
LETTRÉ, H.—(1941) *Z. physiol. Chem.*, **271**, 192.
LOEWENTHAL, H. AND JAHN, G.—(1932) *Z. Krebsforsch.*, **37**, 437.